# Correction

**DOI:** 10.1093/nar/gkae352

**Published:** 2024-04-30

This is a correction to:

J Brooks Crickard, Chaoyou Xue, Weibin Wang, Youngho Kwon, Patrick Sung, Eric C Greene, The RecQ helicase Sgs1 drives ATP-dependent disruption of Rad51 filaments, *Nucleic Acids Research*, Volume 47, Issue 9, 21 May 2019, Pages 4694–4706, https://doi.org/10.1093/nar/gkz186

Chaoyou Xue, James M Daley, Xiaoyu Xue, Justin Steinfeld, Youngho Kwon, Patrick Sung, Eric C Greene, Single-molecule visualization of human BLM helicase as it acts upon double- and single-stranded DNA substrates, *Nucleic Acids Research*, Volume 47, Issue 21, 02 December 2019, Pages 11225–11237, https://doi.org/10.1093/nar/gkz810

Chaoyou Xue, Lucia Molnarova, Justin B Steinfeld, Weixing Zhao, Chujian Ma, Mario Spirek, Kyle Kaniecki, Youngho Kwon, Ondrej Beláň, Katerina Krejci, Simon J Boulton, Patrick Sung, Eric C Greene, Lumir Krejci, Single-molecule visualization of human RECQ5 interactions with single-stranded DNA recombination intermediates, *Nucleic Acids Research*, Volume 49, Issue 1, 11 January 2021, Pages 285–305, https://doi.org/10.1093/nar/gkaa1184

In the Funding section of the above-listed articles, an incorrect grant number is stated for author Eric C Greene (“National Science Foundation Grant MCB-1154511”).

The correct grant number is “National Science Foundation Grant MCB-1817315”.

These details have been corrected only in this correction notice to preserve the published versions of record.

